# The impact of trained patient educators on musculoskeletal clinical skills attainment in pre-clerkship medical students

**DOI:** 10.1186/1472-6920-11-65

**Published:** 2011-09-23

**Authors:** Anna E Oswald, Mary J Bell, Jeffrey Wiseman, Linda Snell

**Affiliations:** 1 Division of Rheumatology, Department of Medicine, University of Alberta, Edmonton, Canada; 2 Division of Rheumatology, Department of Medicine, University of Toronto, Toronto, Canada; 3 Centre for Medical Education and Department of Medicine, McGill University, Montreal, Canada

## Abstract

**Background:**

Despite the high burden of musculoskeletal (MSK) diseases, few generalists are comfortable teaching MSK physical examination (PE) skills. Patient Partners^® ^in Arthritis (PP^®^IA) is a standardized patient educator program that could potentially supplement current MSK PE teaching. This study aims to determine if differences exist in MSK PE skills between non-MSK specialist physician and PP^®^IA taught students.

**Methods:**

Pre-clerkship medical students attended 2-hour small group MSK PE teaching by either non-MSK specialist physician tutors or by PP^®^IA. All students underwent an MSK OSCE and completed retrospective pre-post questionnaires regarding comfort with MSK PE and interest in MSK.

**Results:**

83 students completed the OSCE (42 PP^®^IA, 41 physician taught) and 82 completed the questionnaire (42 PP^®^IA, 40 physician taught). There were no significant differences between groups in OSCE scores. For all questionnaire items, post-session ratings were significantly higher than pre-session ratings for both groups. In exploratory analysis PP^®^IA students showed significantly greater improvement in 12 of 22 questions including three of five patient-centred learning questions.

**Conclusions:**

PP^®^IA MSK PE teaching is as good as non-MSK specialist physician tutor teaching when measured by a five station OSCE and provide an excellent complementary resource to address current deficits in MSK PE teaching.

## Background

Although musculoskeletal (MSK) complaints comprise 12-20% of primary health care visits and are a source of significant health care expenditure and morbidity, MSK physical examination (PE) is often neglected in clinical practice [1-9]. Further, a decline in enrolment in MSK-related specialties and a perceived low confidence and competence level in MSK PE in generalists, has resulted in a shortage of faculty prepared to teach this subject at all levels of medical education [10-14].

Reports from several constituencies have identified inadequacies in the teaching of MSK PE clinical skills [15-22]. A survey of medical students at Harvard University revealed that although the students considered MSK medicine as the third most important topic to their future medical career, only 26% of fourth year students passed a nationally validated written MSK exam [23]. To improve the consistency of MSK teaching, several national and multinational groups have developed MSK undergraduate medical school training standards [15,17,19,22,24-27].

Patient educators have been successful in teaching many aspects of general and system specific physical examination [28-34]. The Pelvic Exam Program is a patient educator initiative that has improved male and female genital examination clinical skills teaching [35-39].

The features of patient educator led teaching are well aligned with several educational learning theories. Patient educator programs were initially grounded in Bandura's social learning theory and bridge both Lave's Situated Learning Theory and Vygotsky's Social Development Theory [40-42]. Lave's Situated Learning Theory emphasizes that knowledge must be presented in authentic contexts in order to help learners move from the periphery to the centre of a "community of practice". As preclinical medical students are just entering the periphery of the medical community of practice, patient educators present a safe yet contextually authentic way to motivate them and impart knowledge. In terms of Vygotsky's Social Development learning theory, patient educators can act as the "more knowledgeable other" to help students gain more and more independence in their performance of accurate MSK physical examination.

The MSK specific patient educator program, Patient Partners^® ^in Arthritis (PP^®^IA), is a centrally coordinated, standardized national program that trains volunteer patient educators with arthritis to teach and evaluate MSK clinical examination skills. Patient educators in this program undergo 100 hours of training. Though originally developed in 1989, this program was introduced in Canada in 1995 and is now active at thirteen of the seventeen Canadian medical schools at the undergraduate, postgraduate and/or continuing medical education levels. This program was introduced at the pre-clerkship level at McGill University in 2004 as an integral part of the teaching program and student evaluations of this teaching have been uniformly strongly positive.

Medical students report that first patient encounters are strong emotional experiences and first patient physical examinations are often described as anxiety provoking and confusing [43]. They also report feeling conflicted around the issue of using patients for their own learning [43]. Others have shown that the early introduction of clinical skills improves medical students' comfort when they start clerkship [44]. We hypothesize that PP^®^IA patient educator led sessions could provide a transition experience and potentially lower the stress related to first patient encounters. These sessions may also alleviate the guilt related to the students' perception that they are using patients for their own learning as the patient educators are acting as true partners in the students' education. These sessions may also improve interest in MSK as an area of future study as previous studies have shown that, in general, the early introduction of clinical skills teaching and improved student self confidence with the subject area may also enhance learning interest in the subject [45,46].

There have been several descriptive studies of the arthritis patient educator programs' effectiveness [47-49] and six previously reported controlled trials of the impact of arthritis patient educator interventions [50-55]. Quantitative assessments of the efficacy of patient educator versus physician MSK PE teaching have yielded mixed results. However, four of six controlled studies used student self-report of clinical skills or single station objective structured clinical exams (OSCEs) to assess student performance [50,51,53,54]. These studies were characterized by unbalanced controls, small sample size or incomplete description of methods. Further, the varying teaching doses provided (anywhere from one to nine hours) and the differing outcomes measured, may have contributed to the lack of uniformity of the findings of even the more rigorous studies.

One study with very rigorous methodology compared a nine hour intervention of PP^®^IA patient educator led versus rheumatologist-led small group clinical examination teaching [52]. This study found near equivalency of the two teaching methods. However, in the current Canadian climate, medical schools are rarely able to provide such extensive rheumatologist-led small group sessions at the undergraduate level [56]. The most recent study used a well designed two station MSK OSCE and showed equivalency in skills scores but slightly higher student instructor ratings for final year medical students in the UK [55]. This is the only study to date that included power calculations, allowing the authors to conclude equivalency between the 2 groups for the two OSCE stations based on scores within 10%.

Several studies have demonstrated that healthy standardized patients can serve as reliable and effective evaluators of medical students' performance of communication skills and professionalism [57,58], general physical examination skills [59,60] and MSK clinical skills [61]. Intra- and inter-rater reliability in OSCE checklist assessments of medical students has been demonstrated for patient educators trained by the PP^®^IA program [47].

## Objectives

The aim of this study is to address the following research questions: (1) Are there differences in the performance of MSK physical exam skills between students taught by PP^®^IA patient educators and those taught by non-MSK specialist physician tutors? (2) Are there differences in the demonstration of interpersonal skills between students taught by PP^®^IA patient educators and those taught by non-MSK specialist physician tutors? (3) Are there differences between PP^®^IA patient educator and non-MSK specialist physician tutor taught students in their stated comfort with MSK clinical skills and their interest in MSK medicine as an area of future study? (4) Are there differences in instructor evaluations for PP^®^IA patient educators and non-MSK specialist physician tutors? For the purpose of this study, interpersonal skills include but are not limited to: establishment of rapport, sensitivity to patient discomfort during examination, elicitation of the effect of arthritis on patients' lives, and the elicitation of patients' perspectives of disease.

## Methods

### Subjects and Setting

Pre-clerkship second year medical students at McGill University in the Introduction to Internal Medicine (IIM) course at two of the four possible hospital sites (herein referred to as hospital site 1 and hospital site 2) were eligible for this study. The IIM is a mandatory course, where students are taught general PE skills over 12 two-hour small group sessions (total 24 hours) by a general or sub-specialist physician tutor. In some cases, where the undergraduate office has difficulty finding one physician who can commit to twelve sessions, a group may be assigned two physician tutors, who share the sessions. None of the physician tutors who taught the course in the year of this study were MSK medicine specialists.

The assignment of students to a hospital site and to small groups of four to six students is performed by the McGill Office of Undergraduate Medical Education. This office uses a computer program that randomly assigns students to hospital site while maintaining an even distribution of male and female students and of Quebec and non-Quebec residents. In cases of outstanding personal or family circumstances, requests for placement at particular sites are accommodated by this office giving a quasi-random allocation. Approximately eight percent of students eligible for this study made placement requests. The IIM course took place in three seven-week cohorts from February to June 2006 and this study involved students in the first two cohorts of the course.

### Intervention

An MSK PE small group session replaced one of the 12 usual IIM general PE small group sessions in the first week of the IIM block. In the first cohort, one of the usual tutor-led small group sessions was replaced with a patient educator-led small group session of equal duration (approximately two hours) at hospital site 2. The physician tutors were instructed not to attend the patient educator led sessions. Concurrently at hospital site 1, one of the physician tutor small group sessions was attended by an arthritis patient from the community. This patient was meant to ensure that both groups of students were exposed to a patient with arthritis-related findings.

In cross-over design, during the second cohort of the IIM course, the patient educators-led sessions for the hospital site 1 small groups and community arthritis patients were present for the usual tutor led small group sessions at hospital site 2.

### Briefings and Consents

A group briefing session was held for all the patient educators by one of the study authors (AO). At that session, the patient educators were each given a copy of the student IIM MSK supplementary handout which explained the reasons for designating one of the IIM sessions to MSK and the specific objectives (Additional File 1: Appendix 1) of the session. This document was reviewed in detail with the patient educators to ensure that the objectives were clear and they were given the opportunity to ask questions. They were also given an abstract that outlined the purpose and nature of the research project. Consents for the patient educators were obtained at this session.

Because of scheduling difficulties, individual briefing sessions for the physician tutors were given by one of the study authors (AO). At each of these meetings, the same information was given, including an identical copy of the student IIM MSK supplementary handout that explained the reasons for designating one of the IIM sessions to MSK and the specific objectives of the session. This document was reviewed in detail with each physician tutor to ensure that the objectives and expectations of the sessions were clear and they were given the opportunity to ask questions. They were also given a copy of a general abstract that outlined the purpose and nature of the research project. Consents for the physician tutors were obtained at these meetings.

All students attended an orientation session for each IIM block on the first day of each cohort. At this session, in addition to general information about the course, each student was given a copy of the IIM MSK supplementary handout, which explained the reasons for designating one of the IIM sessions to MSK and the specific objectives of this session. Consent was obtained from student participants after the course director had left the room.

### Data Collection

At the end of the second week of the IIM block, all students participated in a formative 'teaching' Objective Structured Clinical Examination (tOSCE). As the students had not yet experienced an OSCE at this stage of their training, the general procedure of how this type of exam is conducted and scored was described at the orientation session on the first day of the course. The students were informed that this was a tOSCE and that attendance would be taken as part of the course evaluation, but actual tOSCE scores would not be disclosed to the course coordinator or to their tutors.

Two experts in OSCE evaluation were consulted to provide a reasonable balance in tOSCE design between available resources and reliability standards (C. van der Vleuten and L. Gruppen). As a result, 5-station tOSCE with eight minute examination periods was developed based on a subset of previously tested OSCE stations from the work of Humphrey-Murto et al (2004). The stations included hand, elbow, shoulder, hip and knee physical examination with very short clinical stems.

The tOSCE evaluations were completed by PP^®^IA patient educators who were blinded to teaching group assignments (students were taught by physician tutor or patient educators). Both students and patient examiners were specifically asked not to discuss teaching group assignment during the tOSCE. The patient evaluators acted simultaneously as both patient and evaluator in the OSCE stations. They spent seven minutes in the patient role while the student performed the specified examination, then gave one minute of verbal feedback on things done well and areas for improvement and then spent two minutes completing the checklist with the student out of the room.

The checklists consisted of a four point section on interpersonal skills that was identical in each of the five stations and a ten point section on physical examination techniques that was station specific. The patient educator evaluators were also asked to give an overall rating on a five point Likert scale where one was 'inferior' and five was 'superior.'

Using the published Humphrey-Murto et al OSCE sample group mean and variance data, power calculations were performed for this study [52]. The calculations revealed that 30.5 students were required per group (61 students total) to achieve 80% power to detect a ten percent difference in OSCE scores.

After the tOSCE was over, all students were asked to complete a self-administered questionnaire. Demographics included: any prior formal training in MSK medicine (e.g. prior training in physiotherapy etc) and gender. The questionnaire asked the students to rate their comfort with different aspects of patient communication, MSK PE knowledge and MSK PE skills (Additional File 1: Appendix 2). These questions were posed in a retrospective pre/post style [62]. Specifically, the students were asked to give each item a retrospective rating reflecting how comfortable they felt prior to the MSK teaching session in one column and a current rating reflecting their comfort level at the time of completing the questionnaire in a second column. The rationale behind this approach is that students may not be aware of their initial deficits until they have completed the small group PE teaching sessions and tOSCE experiences. The response options included: not comfortable, somewhat comfortable, comfortable or very comfortable.

A question regarding interest in MSK as an area of future study was also posed in this style with response options: not interested, somewhat interested, interested and very interested. The students were then asked to rate their instructor and several aspects of their experience of the MSK PE teaching session on a four point scale of: strongly disagree, disagree, agree and strongly agree (Additional File 1: Appendix 3). Finally there were questions requesting open-ended comments regarding positive experiences and areas for improvement. During the teaching sessions, qualitative data was collected and analysed by collective case study methods and is reported in a separate publication [63].

### Data Analysis

The primary outcome measures were physical examination and interpersonal skills via the tOSCE and the degree of change in the retrospective pre-post questionnaire overall scores between the groups. Secondary outcome measures were the students' stated comfort and interest in MSK related fields as measured by the degree of change in the retrospective pre-post questionnaire scores between the groups and the group comparisons of the students' ratings of their instructors.

The interpersonal skills score and physical examination skills score for the tOSCE were determined for each station and means with standard deviations were calculated. These scores were analysed by two tailed t-tests for each station where source of teaching (patient educator versus physician tutor) acted as the independent variable. Data from the same group of individuals was exposed to ten tests (interpersonal and physical exam components for each of the five tOSCE stations). The authors applied the Bonferoni correction to address the issue of type 1 error resulting from repeated testing of the same sample. This correction gives a required p value of 0.005 for significance in this part of the study. The alpha reliability for the five station OSCE (total score of 70) was calculated using the IDEAL item analysis package (version 1.1).

Means and standard deviations of the student global ratings were calculated for each group and were compared using a two-tailed t-test. In this case, data was exposed to 5 tests (global score comparison for five stations) and the Bonferoni correction was again applied giving a required p value of ≤ 0.01 for significance in this section of the study. Global rating scores were also correlated with interpersonal skill and physical exam OSCE scores.

The retrospective pre-post questionnaire data was analysed by assigning scores as follows: 1 = not comfortable, 2 = somewhat comfortable, 3 = comfortable and 4 = very comfortable. Means and standard deviations were calculated for both groups. The difference in the means between for the pre and post responses to the questionnaire was also calculated for both groups. An exploratory analysis was conducted where by the difference of the pre and post means for each question was compared between the two groups using paired t tests. As this data is only exploratory, Bonferoni corrections were not applied.

The analysis of the question regarding future interest in pursuing MSK as an area of future study was done in a similar fashion where the following scores were assigned to the response set: 1 = not interested, 2 = somewhat interested, 3 = interested and 4 = very interested.

Finally, instructor rating scores means and standard deviations for each of the fourteen questions were calculated for each group and compared using two tailed t-tests. All statistical analyses were performed using SPSS version 14.

### Ethics

All data was confidentially coded by an identification number and kept in a secure filing cabinet in the principal investigator's locked office. Minimal demographic information was collected and any information that could identify specific individuals was not included in the analysis. This study was funded by an educational grant from Pfizer and ethics approval was obtained from the McGill University Institutional Review Board.

## Results

### Subjects

Eighty-nine students were eligible for the study. All 89 students consented to participate and attended the MSK small group teaching session. Forty-four students were taught by PP^®^IA patient educators and 45 by physician tutors. Seven students did not complete the tOSCE and/or questionnaire due to schedule conflicts. Thus, full tOSCE data was obtained for 83 students (42 patient educator taught and 41 physician tutor taught) and full questionnaire data was obtained for 82 students (42 patient educator taught and 40 physician tutor taught). One of the 82 students who completed the questionnaire reported having significant prior experience with MSK examination and this student was in a physician tutor led group.

### OSCE interpersonal and physical exam scores

Mean scores, standard deviations and p values for the interpersonal skills and physical examination skills for each of the five stations are reported in Tables 1. Mean scores for interpersonal skills component of each tOSCE station for patient educator taught students ranged from 3.31 to 3.62 (maximum score of 4) and for physician tutor taught students ranged from 3.24 to 3.61 with no significant differences between the groups. Means for physical examination skills tOSCE scores ranged from 6.94 to 8.32 (maximum score of 10) for patient educator taught students and 5.98 and 8.56 for physician tutor taught students with the hip station giving the lowest scores and the elbow station giving the highest scores in both groups. There are no significant differences in physical examination tOSCE scores for any of the stations. The hip station physical exam scores are slightly higher in the PP^®^IA led groups but are not significant after considering the Bonferoni corrected alpha of 0.005. The alpha reliability for the five station OSCE was 0.67 and the standard error of measurement (SEM) was 4.01. Power calculations were repeated using the standard deviations actually obtained in the current study. The sample size necessary to detect a 10% difference in scores with 80% power in the IPS data is 64 and for the physical exam scores is 47 students which are both well below the sample size in the current study.

**Table 1:** OSCE: Interpersonal skills scores, physical examination skills scores and global ratings

Station	Patient educator group mean score (sd)	Physician tutor group mean score (sd)	P value
**Interpersonal Skills**	**Total Score = 4** **n = 42**	**Total Score = 4** **n = 41**	**(α = 0.005 with Bonferoni correction)**

Hand	3.33 (0.87)	3.41 (0.71)	0.643
Shoulder	3.31 (0.84)	3.61 (0.59)	0.063
Elbow	3.43 (0.86)	3.41 (0.77)	0.938
Knee	3.62 (0.62)	3.43 (0.87)	0.282
Hip	3.48 (0.83)	3.24 (1.09)	0.278

**Physical Examination**	**Total Score = 10** **n = 42**	**Total Score = 10** **n = 41**	**(α = 0.005 with Bonferoni correction)**

Hand	7.73 (1.53)	7.66 (1.78)	0.853
Shoulder	7.83 (1.93)	8.12 (2.18)	0.525
Elbow	8.32 (1.48)	8.56 (1.43)	0.456
Knee	7.55 (1.38)	7.58 (1.54)	0.936
Hip	6.94 (1.86)	5.98 (2.02)	0.026

**Global Ratings**	**Total Score = 5** **n = 42**	**Total Score = 5** **n = 41**	**(α = 0.01 with Bonferoni correction)**

Hand	4.05 (0.54)	4.12 (1.38)	0.75
Shoulder	3.95 (0.83)	3.56 (1.14)	0.08
Elbow	4.05 (0.91)	3.95 (0.50)	0.55
Knee	4.52 (1.42)	4.56 (1.83)	0.92
Hip	3.69 (1.18)	3.63 (0.97)	0.81

sd = standard deviation OSCE = objective structured clinical examination

### The tOSCE evaluator global ratings

The mean scores tOSCE evaluator's global ratings of the students with standard deviations for each station are listed by group in Table 1. There are no significant differences between the groups. Global assessment scores for the hand, elbow, shoulder and hip stations all show weak to moderate correlations with both interpersonal skills

(IPS) tOSCE scores and physical examination tOSCE scores (Table 2). The physical examination scores consistently have slightly stronger correlations with global assessment scores than the IPS scores. Correlations with physical exam scores range from -0.11 to 0.64 and correlation with IPS scores ranging from -0.03 to 0.39 (Table 2).

**Table 2:** Correlations between interpersonal skills and physical exam OSCE scores with global assessment OSCE scores

Station	Total group mean IPS score (sd) (1 = Inferior 5 = Superior) n = 83	Total group mean Physical exam score (sd) (1 = Inferior 5 = Superior) n = 83
Hand	0.35	0.44
Shoulder	0.23	0.51
Elbow	0.39	0.41
Knee	- 0.03	- 0.11
Hip	0.26	0.64

IPS = Interpersonal Score

sd = standard deviation

OSCE = objective structured clinical examination

### Retrospective pre-post questionnaire: Student comfort level

For all 22 questions relating to comfort with different aspects of the MSK PE, the post session ratings were significantly higher than the "pre session" ratings for both patient educators led students and physician led students. Patient educator led students showed more improvement in comfort levels post session than those in the physician tutor led groups in all cases. When exploratory analysis was performed, these differences were found to be significant in twelve of the 22 questions as listed in Table 3. These include three of the five patient centred learning related questions.

**Table 3:** Retrospective questionnaire: exploratory comparison of differences in mean pre - post scores between student groups (see Additional File 1: Appendix 2 for complete list of questions)

Question	Patient educator group mean differences post - pre score (sd) Maximum difference = 3 n = 42	Physician tutor group mean differences post-pre score (sd) Maximum difference = 3 n = 40	P value
*1. Overall techniques of MSK examination*	1.10 (0.76)	0.70 (0.69)	0.016
*2. Inspection for erythema, swelling & deformity*	0.88 (0.76)	0.43 (0.64)	0.004
*3. Performing active ROM*	0.86 (0.78)	0.43 (0.50)	0.004
*10. Overall approach to elbows*	1.02 (0.78)	0.93 (1.39)	0.014
*11. Overall approach to shoulders*	1.10 (0.85)	0.55 (0.64)	0.002
*12. Overall approach to hips*	0.98 (0.81)	0.60 (0.63)	0.022
*14. Overall approach to feet*	0.83 (0.85)	0.43 (0.50)	0.010
*15. Identifying normal vs. abnormal*	0.98 (1.07)	0.60 (0.55)	0.050
*17. Identifying when special physical exam manoeuvres should be done*	0.79 (1.07)	0.35 (0.53)	0.023
*19. Identifying how lives of patients with MSK concerns may be affected*	1.19 (1.11)	0.40 (0.71)	0.000
*20. Demonstrating concern for patient comfort during MSK exam*.	1.05 (1.55)	0.43 (0.90)	0.000
*21. Using feedback from a patient I'm examining to further my learning*	1.12 (1.49)	0.50 (0.82)	0.023

sd = standard deviation

MSK = Musculoskeletal

ROM = range of motion

### Retrospective pre-post questionnaire: Student interest in MSK

Both patient educator led and physician led student groups showed significant increase in interest MSK as an area of further reading, electives or training, after the teaching and tOSCE experiences (p = 0.000 and 0.001 respectively) but there was no significant difference between the two groups (p = 0.25).

### Instructor evaluations

For the fourteen instructor evaluation questions, scores range from 2.79 to 3.95 for the PP^®^IA patient educator led group compared to 2.85 to 3.68 for the physician led group (maximum possible score of 4). For ten of the fourteen questions, scores were significantly higher for the patient educator led group compared to the physician led group with the remaining questions showing no significant differences (Table 4). The greatest differences between the groups were seen in session relevance (question 13, difference in score of 0.64) and ensuring patients' comfort during the examination (question 7, difference in score of 0.44).

**Table 4:** Instructor evaluation scores (see Additional File 1: Appendix 3 for specific question wording)

Question Topic	Patient educator group mean score (sd) Max score = 4 n = 42	Physician tutor group mean score (sd) Max score = 4 n = 40	P value
1. Provided general approach	3.20 (0.51)	3.20 (0.82)	0.069
2. Treated students with respect	3.93 (0.26)	3.68 (0.73)	0.043
3. Safe & open learning environment	3.95 (0.22)	3.58 (0.75)	0.004
4. Encouraged questions	3.90 (0.30)	3.60 (0.74)	0.019
5. Answers to questions helpful	3.76 (0.43)	3.48 (0.78)	0.046
6. Emphasized how arthritis affects patients	3.62 (0.54)	3.28 (0.85)	0.030
7. Emphasized ensuring patient comfort	3.71 (0.77)	3.28 (0.82)	0.014
8. Demonstrates techniques clearly	3.69 (0.47)	3.30 (0.85)	0.013
9. Demonstrates normal vs. abnormal	3.10 (0.76)	3.28 (0.78)	0.295
10. Demonstrates inflammatory vs. degenerative	2.79 (0.84)	2.85 (0.89)	0.738
11. Allow time for practicing	3.29 (0.71)	2.90 (0.84)	0.027
12. Gives constructive feedback	3.43 (0.59)	3.23 (0.83)	0.203
13. Makes relevance of session clear	3.69 (0.52)	3.05 (1.01)	0.000
14. Makes session interesting	3.79 (0.42)	3.43 (0.75)	0.009

sd = standard deviation

## Discussion

This appropriately powered study demonstrates that there are no significant differences in MSK physical examination OSCE scores or interpersonal skills OSCE scores between students taught by trained patient educators and those taught by usual non-MSK specialist physician tutors. This study adds to the current patient educator literature in that it provides rigorous evidence for a much more practical approach to integrating arthritis patient educators using the currently employed standardized PP^®^IA program. It evaluates the effectiveness of a much more realistic and generalize-able intervention, that being a brief patient educator teaching session, and uses a much more realistic and generalize-able comparator, that being non-MSK specialist physician tutors than has been reported in previous literature. This real-life intervention is supported by recent surveys of actual practices in MSK clinical skills teaching in Canada [56].

This brief teaching session was also successful in improving students' retrospective pre and post ratings of comfort levels with many different aspects of MSK examination. It is reassuring that both groups' perceptions of comfort improved after the teaching and tOSCE sessions. It is also interesting that where significant differences did exist between the groups, PP^®^IA patient educator led students consistently reported more improvement in their comfort levels than did physician tutor led students. With such a short teaching intervention, it is probably not surprising that significant differences in stated interest in further study in MSK were not seen.

One of the two rigorously conducted previous studies also showed PP^®^IA patient educator teaching can result in similar OSCE scores for a nine hour teaching intervention compared to a similar duration session given by rheumatologists [52]. However, this study cannot comment on actual equivalence, as power calculations were not presented. Previously published surveys show that this amount of either patient educator led small group time or specialist-led small group time far exceeds what the vast majority of schools currently provide [3,56,64,65]. The current study's findings extend the results of Humphrey-Murto's study to a shorter teaching intervention of two hours total duration, which is more realistic in the preclinical setting. This study also extends these findings from MSK specialist physician tutors to general physician tutors. This is very relevant as survey data documents that non-MSK specialist clinical teachers teach MSK clinical skills at 60% of Canadian medical schools [56].

Although the other rigorously conducted study did present power calculations and did find equivalence between patient educators for a 2 hours intervention, it also used rheumatologists as its comparison, which again is not representative usual teachers of MSK clinical skills in most universities. In addition, this study did not use the existing standardized patient educator training program (PP^®^IA), but rather employed its own unique training strategy to prepare patient educators which further limits the ability to generalize findings to other centres [55].

Unlike the Humphrey-Murto study, there were no tOSCE stations showing significant differences between the patient educators and physician tutor groups in the current study. One potential consideration that may account for this is the fact that the two stations where they found significant differences (ankle examination and sciatica examination) were not included in our tOSCE. The objectives for our teaching sessions were clear in their inclusion only of major peripheral joints. When considering the short time available, the authors did not feel that both the spine examination and the complete peripheral joint examination could be meaningfully covered in a two hour teaching session. However, the differences that they found were both in favour of the rheumatologist taught students. In our study, the hip station was the only one that came even close to significance and unlike the previous study, this was in favour of the patient educator taught students. It must be noted, however, that the current study focuses on peripheral joint exam and these finding may not be extrapolated to other aspects of MSK examination such as spine, soft tissue, power or gait examinations.

There were only five tOSCE stations used in our study compared to nine stations in Humphrey-Murto's study. When determining the numbers of stations to include in our tOSCE, it was important to consider teaching "dose" versus examination duration. Since only two hours of teaching were provided in this study, it was difficult to justify a nine station tOSCE. In attempts to resolve this dilemma, the author consulted two international experts in clinical skills evaluation (CV and LG). On considering their advice, it was decided that a five station OSCE would give the best balance of reliability through number of stations and sampling validity through an appropriate teaching to examination ratio.

There are several limitations that need to be considered when interpreting the results of this study. They include potential issues relating to sampling validity, sensitivity of the outcome measures and external validity.

Firstly, one must consider how representative the sample studied is compared to the general cohort of both instructors and students. As part of the criteria for the study, none of the physician tutors were specialists in MSK disciplines such as rheumatology, orthopedic surgery or physiatry. In all cases, the tutor agreed to give this MSK session. This reduced the possibility that participating tutors were more or less skilled at teaching MSK PE as all were included. Some of the participating tutors did express that they had not taught MSK PE when they taught this course in the past. This would indicate that we were not dealing with a group of general physician tutors that were unusually confident in their MSK teaching. The patient educators who participated in the teaching volunteered to give three two-hour teaching sessions over the 21 week IIM course. Although it could be argued that those who volunteered may have been more committed to the program, this type of selection would be parallel to what would occur if patient educators were asked to participate in actual undergraduate teaching sessions of similar duration. In terms of the students, student assignment to hospital site was quasi-random through the central Undergraduate Education Office and the few requests for change in placement occurred prior to students' knowledge of the study. Thus, it is unlikely that these requests would have caused systematic differences between these students and those at the other teaching hospitals.

Another potential question to the validity of the study relates to whether the tOSCE tested material that was representative of what students should learn in an undergraduate MSK PE session. The OSCE was structured around course objectives that were derived from the literature review on undergraduate MSK teaching objectives. One may wonder if a tOSCE is sensitive enough to pick up relevant differences between the intervention groups. However, a similar OSCE did pick up differences in the previous study by Humphrey-Murto and the reliability of 0.67 for the current study's OSCE was similar and high according to Cohen's guidelines [66]. One may also question whether the seven to ten day delay between the teaching and the tOSCE was appropriate. We chose this timing to minimize the influence of confounders on the results but we recognize that future studies with a second delayed OSCE to evaluate long-term retention would be desirable.

One may also wonder if the novelty of this intervention created a Hawthorne effect. However, as there was a change to the normal structure of both groups by way of the presence of a community arthritis patient or a patient educator, and as no differences were found between the groups this is unlikely to have had a major confounding effect. Had it done so, one would expect that the patient educator group would have outperformed the physician led group and this was not the case. One may also question the validity of student self-assessment. However, this study does not draw conclusions on the absolute self-confidence ratings but rather evaluates the comparison between two quasi-randomly selected groups of students. There is not reason to believe that there would be differences in the reliability of this data between the two groups.

Finally one may question whether the findings from a study performed at one University in one country can be generalized more widely. Fortunately, when comparing this university's practice to those reported in national Canadian, American and UK surveys, the amount of teaching offered appears more in line with standard MSK teaching practice in that a very brief amount of MSK clinical skills teaching is offered, it is offered primarily by non-MSK specialist physicians and is supplemented by patient educators [3,56,64,65].

There are many interesting points raised by this study. For example, the authors find it somewhat unusual that there were no differences in physical examination skills tOSCE scores despite reports from the qualitative data referred to earlier in this paper that some physician tutors did not manage to cover all of the specified joints in their teaching sessions [63]. These findings may be explained by the fact that students were aware of the upcoming OSCE and may have read independently to bring their knowledge in par with objectives regardless of teaching content or source. Although assessment is known to drive learning, previous studies with non-teaching comparison arms have demonstrated significant differences in MSK PE skills even when students were aware of an upcoming tOSCE [37,38].

In a qualitative analysis that occurred in parallel to this study, the authors found that physician tutors were more likely to emphasize the verbalization of physical exam manoeuvres as they were performed, than patient educators [63]. Differences in the students' abilities to express rather than perform skills could have led to a bias in the results in favour of the physician tutor group. Despite this, no significant differences were found.

The early introduction of patients into the undergraduate medical curriculum is desirable as it may improve the authenticity of the teaching experience by making the context of the learning environment similar to what students will experience in their future clinical roles. Patient educators give students experience on how to conduct themselves in front of real patients and give them more responsibility to develop rapport with patients who can give specific feedback in the absence of supervising physicians. Furthermore, patient educators provide students with explicit opportunities to learn from patients, appreciate patients as knowledgeable partners, and incorporate the patient's perspective as part of the educational messages.

The development of strong MSK PE skills is important for accurate diagnosis of common MSK complaints in both primary and specialty based patient care. As discussed in the introduction, MSK conditions comprise a significant proportion of current health care visits and this is likely to be more important in the future with the aging of the population. The initial reluctance of the physician tutors to include an MSK PE session in their usual teaching in this undergraduate clinical skills course underscores the fact that this teaching was likely not consistently occurring prior to this study.

## Conclusions

This study supports the use of PP^®^IA patient educators as a teaching tool in undergraduate curriculum in conjunction with physician tutor teaching. It extends previous findings of minimal difference between rheumatologist and patient educators in pre-clerkship teaching to a more realistic scenario of a two hour MSK teaching session embedded within a general clinical skills teaching course given by non-MSK specialist physicians. Course directors and curriculum planners could honour and augment the scarce physician tutor resource by employing physician tutors more efficiently once students have chance to develop an approach to the basic skills of joint exam through patient educator led teaching sessions.

## Competing interests

A representative from Pfizer^®^Canada has seen the final manuscript but no company representative had any part in the development of the study design, data collection, data analysis or manuscript preparation for this study. There are no other competing interests.

## Authors' contributions

AEO was the primary person responsible for developing the study design, completing the data collection, analysing the data and writing the manuscript. JW contributed significantly to the study design, assisted with data collection and edited the manuscript. MJB contributed to the study design and significantly edited the manuscript. LS contributed significantly to the study design, assisted with data collection and edited the manuscript. All authors read and approved the final manuscript.

## Pre-publication history

The pre-publication history for this paper can be accessed here:

http://www.biomedcentral.com/1472-6920/11/65/prepub

## Supplementary Material

Additional file 1 **Appendices**. Appendices 1, 2 and 3. Click here for file
