# Supplementary material for: The impact of trained patient educators on musculoskeletal clinical skills attainment in pre-clerkship medical students
Source: BMC Med Educ. 2011 Sep 23;11:65. doi: 10.1186/1472-6920-11-65 (PMC3190339; doi:10.1186/1472-6920-11-65)
Supplement: Additional file 1 — Appendices. Appendices 1, 2 and 3. [file 1472-6920-11-65-S1.DOC]

Appendices

**Appendix 1 - Objectives table provided to students, patient educators and physician tutors prior to the MSK teaching session.**

What should you try to get out of this session?

| **Aim** | **Objective** | **Have I Done This?** |
| --- | --- | --- |
| A. **Technical** aspects of MSK **Examination** including:  🡪 Inspection (erythema, swelling & deformity)  🡪 Active Range of Motion  🡪 Passive Range of Motion  🡪 Palpation (temperature, soft tissue and bony structures, effusions)  🡪 Common “Special Tests”  (= physical manoeuvres to help Dx specific problems) | To perform physical examination of the upper extremity joints (hand, wrist, elbow and shoulder) | ___ Hand?  ___ Wrist?  ___ Elbow?  ___ Shoulder? |
|  | To perform physical examination of the lower extremity joints (foot, ankle, knee and hip) | ___ Foot?  ___ Ankle?  ___ Knee?  ___ Hip? |
| B. Use of **Clinical Reasoning** in MSK exam | To interpret MSK findings as normal or abnormal | ___ |
|  | To identify abnormal findings as more likely degenerative or inflammatory changes | ___ |
|  | To identify when additional physical manoeuvres needed | ___ |
| C. **Communication, Attitude and Empathy** behaviours in MSK physical exam | To demonstrate proficiency in communicating with patients with MSK concerns | ___ |
|  | To demonstrate knowledge of how the lives of patients with MSK problems may be affected by these changes | ___ |
|  | To demonstrate concern for patients’ comfort during examination | ___ |

**Appendix 2 - Retrospective pre-post questionnaire provided to all students immediately following the OSCE**

Please rate your comfort with the following skills considering how you would have felt ***BEFORE*** the musculoskeletal (MSK) teaching session versus ***AFTER*** the MSK teaching session. **NC = Not comfortable; C = Comfortable**

**SC = Somewhat comfortable VC = Very comfortable**

|  | **BEFORE** MSK Teaching | | | |  | **AFTER** MSK Teaching | | | |
| --- | --- | --- | --- | --- | --- | --- | --- | --- | --- |
|  | **NC** | **SC** | **C** | **VC** |  | **NC** | **SC** | **C** | **VC** |
| 1. Overall techniques of MSK examination |  |  |  |  |  |  |  |  |  |
| 2. Inspection for erythema, swelling & deformity |  |  |  |  |  |  |  |  |  |
| 3. Performing active range of motion |  |  |  |  |  |  |  |  |  |
| 4. Performing passive range of motion |  |  |  |  |  |  |  |  |  |
| 5. Palpating for temperature, soft tissue structures and bony structures |  |  |  |  |  |  |  |  |  |
| 6. Palpating for joint effusions |  |  |  |  |  |  |  |  |  |
| 7. Performing common special tests (= physical exam manoeuvres) |  |  |  |  |  |  |  |  |  |
| 8. Overall screening MSK examination |  |  |  |  |  |  |  |  |  |
| 9. Overall approach to hands & wrists |  |  |  |  |  |  |  |  |  |
| 10. Overall approach to elbows |  |  |  |  |  |  |  |  |  |
| 11. Overall approach to shoulders |  |  |  |  |  |  |  |  |  |
| 12. Overall approach to hips |  |  |  |  |  |  |  |  |  |
| 13. Overall approach to knees |  |  |  |  |  |  |  |  |  |
| 14. Overall approach to feet |  |  |  |  |  |  |  |  |  |
| 15. Identifying normal vs. abnormal |  |  |  |  |  |  |  |  |  |
| 16. Identifying findings as more likely degenerative or inflammatory |  |  |  |  |  |  |  |  |  |
| 17. Identifying when special physical exam manoeuvres should be done |  |  |  |  |  |  |  |  |  |
| 18. Communicating with patients with MSK problems |  |  |  |  |  |  |  |  |  |
| 19. Identifying how lives of patients with MSK concerns may be affected |  |  |  |  |  |  |  |  |  |
| 20. Demonstrating concern for patient comfort during MSK examination |  |  |  |  |  |  |  |  |  |
| 21. Using feedback from a patient I’m examining to further my learning |  |  |  |  |  |  |  |  |  |
| 22. Interacting with patients who take an active role in their medical care |  |  |  |  |  |  |  |  |  |

**Appendix 3 - Instructor evaluation form provided to all students immediately following the OSCE**

**Please rate your instructor (tutor or patient partner) on the following questions:**

|  | **Strongly Disagree** | **Disagree** | **Agree** | **Strongly Agree** |
| --- | --- | --- | --- | --- |
| 1. Provided a general approach to the MSK examination. |  |  |  |  |
| 2. Treated students with respect. |  |  |  |  |
| 3. Created a safe and open learning environment. |  |  |  |  |
| 4. Encouraged questions from the students. |  |  |  |  |
| 5. Answers to my questions were helpful. |  |  |  |  |
| 6. Emphasized importance of finding out how arthritis has affects a patient's life. |  |  |  |  |
| 7. Emphasized how to ensure patient's comfort during the examination. |  |  |  |  |
| 8. Demonstrated techniques clearly. |  |  |  |  |
| 9. Demonstrated how to differentiate normal vs. abnormal findings. |  |  |  |  |
| 10. Demonstrated how to differentiate inflammatory vs. degenerative findings. |  |  |  |  |
| 11. Allowed adequate time for students to practice doing the examinations. |  |  |  |  |
| 12. Gave students constructive feedback on their technique. |  |  |  |  |
| 13. Made it clear why the session is relevant. |  |  |  |  |
| 14. Made the session interesting. |  |  |  |  |

**17. Things the instructor did that were helpful or that I would like them to do again next time:**

**18. Things the instructor could improve upon for next time:**
